# Real-time digital monitoring of continuous bladder irrigation: clinical evaluation of a sensor-based system for hematuria and catheter-associated events

**DOI:** 10.3389/fdgth.2025.1634537

**Published:** 2025-11-25

**Authors:** M. Glienke, A. Krumnau, A. C. Reichelt, G. Reis, C. Steiner, P. F. Pohlmann, F. F. Dressler, C. Gratzke, A. Miernik, D. S. Schöb

**Affiliations:** 1 Department of Urology, Faculty of Medicine, Medical Centre—University of Freiburg, Freiburg, Germany; 2 German Research Center for Artificial Intelligence, Kaiserslautern, Germany; 3 Digital Biomedical Imaging Systems AG, Pforzheim, Germany; 4 Department of Pathology, Faculty of Medicine, Charité—Universitätsmedizin Berlin, Berlin, Germany

**Keywords:** bladder irrigation, hematuria, catheter monitoring, digital health, urology, real-time sensoring, patient safety, sensor technology

## Abstract

**Introduction:**

Continuous bladder irrigation (CBI) is commonly applied after transurethral resection of the prostate (TURP) or bladder tumor (TURBT) to prevent clot formation and maintain catheter patency. Despite its widespread use, the monitoring of CBI remains largely manual and subjective, relying on intermittent visual inspection of outflow characteristics. This approach is labor-intensive, prone to inter-observer variability, and can delay recognition of complications such as active bleeding, catheter obstruction, or bladder overdistension. We developed VisIMon, a digital monitoring system that enables continuous, sensor-based surveillance of CBI parameters including hemoglobin (Hb) concentration, inflow/outflow volumes, and flow disturbances.

**Methods:**

In this prospective feasibility study, 20 patients undergoing CBI after transurethral surgery were monitored with the VisIMon system for approximately four hours postoperatively. The system continuously recorded Hb concentration in the outflow via an optical sensor and tracked fluid dynamics using weight-based measurements. Manual reference data were collected every 20 min using a graduated measuring beaker and a digital scale. Blood gas analysis (BGA) was performed at the clinician's discretion in cases of suspected bleeding. All data were synchronized and visualized for graphical analysis.

**Results:**

The system operated reliably in all patients without technical failure or adverse events. The mean deviation between VisIMon-based and manually measured outflow volumes was −16 ml (range: −84 to + 73 ml), indicating acceptable accuracy. Hb trends recorded by the sensor corresponded qualitatively with available BGA data. The system detected clinically relevant flow irregularities—such as drainage interruptions and air bubbles—which were confirmed during bedside assessments. Patients tolerated the system well, and staff reported high usability and value in the graphical displays of real-time irrigation dynamics.

**Conclusion:**

The VisIMon system enabled continuous, objective monitoring of bladder irrigation in postoperative urological care. It demonstrated feasibility, accuracy, and user acceptance in a clinical setting and offers a promising tool for improving patient safety and workflow efficiency. Further validation in larger studies is warranted to assess its long-term impact and potential integration into closed-loop irrigation systems.

## 1 Introduction

Transurethral surgical procedures, such as transurethral resection of the prostate (TURP) or bladder tumor resection (TURBT), are among the most frequently performed interventions in urology. Despite continuous advancements in surgical techniques and perioperative care, catheter-associated complications remain common in the immediate postoperative period. These include hematuria, clot formation, catheter obstruction, bladder overdistension, and, in severe cases, re-intervention or urosepsis (1, 2). To mitigate these risks, continuous bladder irrigation (CBI) is routinely applied to maintain catheter patency, prevent tamponade, and allow early recognition of bleeding or mechanical dysfunction (3).

In current clinical practice, the monitoring of CBI relies almost exclusively on manual and subjective assessment. Medical staff visually evaluate the color and volume of the irrigation outflow, estimate bleeding severity using vague descriptors such as “light” or “severe,” and adjust the irrigation flow based largely on personal experience. This approach is highly variable, prone to inter-observer inconsistency, and places a significant burden on nursing resources. Moreover, timely detection of complications—such as impaired drainage, unexpected changes in inflow or outflow, or subtle shifts in bleeding patterns—can be delayed, particularly during night shifts or in high-patient-load settings (4). Several studies have attempted to address the issue of inter-observer variability by developing standardized visual scales to describe the degree of hematuria more objectively. Notably, Wong et al. (2010) proposed and validated a macroscopic hematuria scale aimed at improving communication and creating an objective hematuria index (5). Lee et al. (2013) introduced a hematuria grading scale as a new tool to classify gross hematuria (6), while Stout et al. (2021) developed a visual scale specifically designed to facilitate communication regarding gross hematuria in clinical settings (7). Despite these promising efforts, none of these scales have been widely adopted in routine clinical practice, underscoring the ongoing need for practical, standardized, and potentially automated solutions.

Moreover, current CBI systems offer no built-in mechanism to detect mechanical issues such as catheter kinking or obstruction, nor do they provide continuous information on bladder filling or fluid balance. These gaps pose serious safety risks: unnoticed overfilling can lead to drainage failure and result in acute urinary retention or clot formation (8). Findings from our recent study evaluating functional parameters, patient-reported outcomes, and nursing workload during continuous bladder irrigation after transurethral surgery underscore these concerns. In clinical practice, episodes of catheter occlusion, unnoticed bladder distension, and fluid imbalance were not uncommon and often led to increased manual interventions and staff workload. Importantly, data also suggest that patients are generally open to the use of electronic monitoring systems during CBI. Such systems were perceived not as intrusive, but as supportive elements of care, potentially enhancing confidence in the clinical process (9). These results highlight the pressing need for more reliable, automated monitoring tools. An integrated, real-time system capable of detecting both mechanical disturbances and changes in bleeding severity could not only enhance patient safety but also alleviate staff burden and improve overall care efficiency in the postoperative setting.

To address this clinical need, we developed an integrated digital sensor system for continuous CBI monitoring. The system includes an optical sensor for hemoglobin (Hb) detection in the irrigation outflow and weight sensors to track inflow and outflow volumes, and algorithms to assess potential mechanical disturbances such as catheter obstruction or air bubbles (10).

This study represents the first clinical application of the sensor-based system in patients undergoing transurethral urological procedures. Our primary objective was to evaluate the feasibility and safety of the device during routine postoperative CBI. Secondary aims included assessing the system's accuracy in measuring Hb concentration and fluid volumes, its ability to detect catheter-associated events, and its usability in a real-world clinical setting. We hypothesize that this digital solution may enhance patient safety, optimize workflow, and pave the way for more objective, data-driven postoperative care.

## 2 Methods

This prospective feasibility study was conducted at the Department of Urology, University Medical Center Freiburg, and was approved by the institutional ethics committee, 20-1177 MPG §23b, 29 October 2020. The study was registered according to article 35 of the Declaration of Helsinki 2013 at Deutsches Register Klinischer Studien (DRKS) under ID DRKS00023647. The study was designed and reported in accordance with the CONSORT 2010 extension for pilot and feasibility trials to ensure transparency and methodological rigor (11).

A total of 20 patients undergoing CBI following transurethral procedures were enrolled after informed consent was obtained. The sample size of 20 patients was pragmatically chosen in line with established guidance for pilot and feasibility studies, which typically recommend 12–30 participants to assess technical feasibility, usability, and data quality without requiring a formal power calculation (12, 13). Although the sensor system had not yet received MDR certification at the time of the study, all necessary risk mitigation measures were implemented, including thorough preclinical testing, continuous on-site supervision, and real-time monitoring. The number of enrolled participants was determined in consultation with the institutional ethics committee and was considered sufficient to evaluate system functionality and monitoring stability across a representative range of postoperative scenarios. The study was not designed or powered to detect statistically significant clinical differences between groups.

Patients were recruited between January and March 2021. Eligible patients were identified during preoperative assessment and informed about the study during routine counseling as part of the admission process. Consecutive patients scheduled for TUR-P or TURBT with subsequent CBI were considered for inclusion. Inclusion criteria were: age ≥18 years, planned transurethral urological procedure with postoperative CBI, written informed consent in accordance with international guidelines and national legislation, and sufficient comprehension of the study procedures. Patients were excluded if they were unable to give consent, had contraindications to CBI, or were enrolled in conflicting interventional trials.

**Figure 1:**
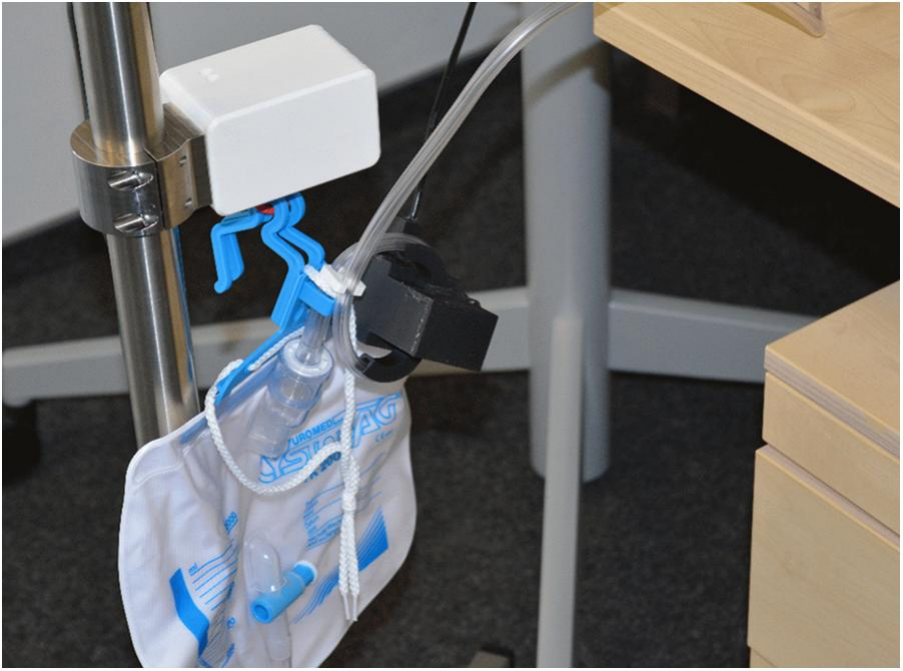
Setup of the VisIMon system during clinical application. The optical sensor with integrated camera module is positioned in front of the drainage bag to enable continuous real-time analysis of the drainage fluid's color characteristics for hemoglobin (Hb) concentration estimation. Weight sensors attached to the irrigation and drainage bags allow simultaneous measurement of inflow and outflow volumes. All parameters were visualized in real time via the system's graphical user interface.

The VisIMon system, a digital, sensor-based monitoring platform, was used for real-time surveillance of CBI during the immediate postoperative period. The system was designed to improve the detection of catheter-associated complications and enhance objectivity in monitoring bleeding severity. It combines several sensor modalities to continuously monitor irrigation parameters and detect clinically relevant events. Inflow volumes were measured using a weight sensor attached to the irrigation bag, while outflow volumes were recorded via a weight sensor on the drainage bag, ensuring precise quantification of flow rates and real-time fluid balance. An optical sensor with an integrated camera system simultaneously analyzed the color characteristics of the drainage fluid to estimate Hb concentration (Figure 1). The optical sensor was calibrated prior to clinical use using a laboratory-grade hemoglobin analyzer (HiCN reference method; measurement range: 0–3.2 g/dL; within-run CV < 1%). During the clinical study, the system continuously recorded hemoglobin estimates at a sampling rate of 10 Hz. BGA was performed on the irrigation outflow fluid, selectively initiated by the clinical team in response to suspected bleeding or abnormal sensor readings. Although non-invasive, frequent sampling would have significantly increased clinical workload. Moreover, standardizing BGA at long fixed intervals (e.g., every 20 min) risks missing relevant bleeding episodes. Therefore, a targeted sampling strategy was used in this feasibility context. On the basis of this data, an embedded algorithm detected deviations in flow patterns and volume discrepancies, which could indicate catheter obstruction, air entrapment, or changes in bleeding severity.

**Figure 2:**
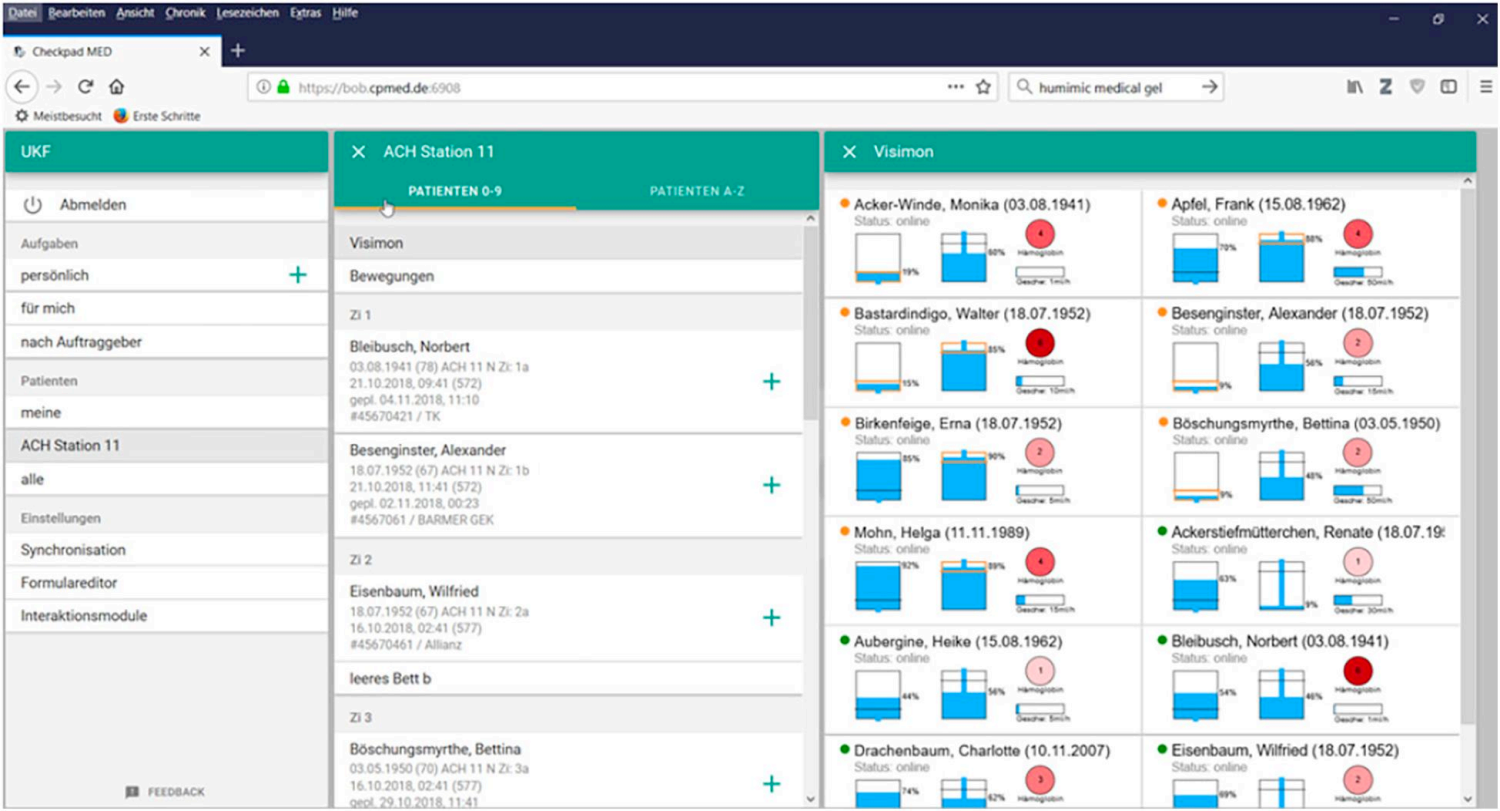
Graphical user interface of the VisIMon system as displayed on a central workstation or tablet device. The interface shows real-time data including the weight of the irrigation and drainage bags and the hemoglobin (Hb) concentrations measured for different patients. The names and birthdates shown in the screenshot are for visualization purposes only and do not correspond to any real patient data.

The technical principles and the underlying algorithmic approach have been previously described by Reis et al. (2021) (10). To enhance transparency and facilitate reproducibility, we briefly summarize the core components of the embedded signal-processing pipeline applied in this study. The system continuously processes synchronized input from three sensor modalities: a load cell beneath the irrigation bag for inflow measurement, a second load cell beneath the drainage bag for outflow quantification, and an optical photometric sensor integrated into the drainage tube for hemoglobin estimation. Raw weight data from both flow channels are smoothed using a five-second moving average filter to suppress high-frequency noise caused by patient movement or ambient mechanical disturbances. Based on these smoothed signals, the algorithm calculates real-time flow rates and monitors the cumulative fluid balance. Deviations in flow behavior are classified through predefined signal thresholds. Catheter stagnation is identified when outflow decreases by more than 70% within a two-minute window relative to the preceding flow average. Air entrapment is recognized by low-amplitude oscillations in the outflow signal combined with brief, non-harmonic fluctuations in the photometric signal, which typically reflect air bubbles passing the optical sensor. Volume discrepancy alerts are triggered when the mismatch between inflow and outflow exceeds 50 mL within a ten-minute rolling interval, potentially indicating catheter occlusion, irrigation leakage, or incorrect bag positioning. Hemoglobin concentration in the drainage fluid is estimated via dual-wavelength photometry at 540 and 575 nm. The system applies the Beer–Lambert law to determine relative absorbance changes and continuously compares these to a baseline established during the initial irrigation phase. Rather than delivering absolute hemoglobin values, the algorithm identifies relative increases over time, which may indicate progressive bleeding or rebleeding events. This trend-based approach was deliberately chosen to minimize the influence of patient-specific optical variability and lighting conditions. In addition to the rule-based signal detection, a convolutional neural network (CNN) is integrated into the optical analysis pipeline. This model was trained on labeled color images of drainage fluid to classify fluid coloration into clinically relevant categories, including clear fluid, light hematuria, and macroscopic hematuria. The CNN output is used to support and verify the trend detection based on photometric absorbance, adding an additional layer of classification that is robust against motion artifacts and lighting variations. All detected events are automatically time-stamped and visualized in real time through the system's graphical user interface (Figure 2). The GUI displays dynamic flow curves, colorimetric signal profiles, and event annotations to support continuous monitoring. All data are locally stored and can optionally be exported in HL7 or FHIR-compatible formats for integration into electronic health records or clinical documentation systems.

In the current study, the VisIMon system was applied for a monitoring period of approximately four hours postoperatively. Sensor-derived measurements were validated against conventional manual methods. Irrigation outflow volume was documented using two reference standards: volumetric measurement via a graduated collection container and weighing of drainage output every 20 min using a calibrated digital scale. In selected cases, where the system indicated relevant changes in hematuria severity, additional blood gas analyses (BGA) were performed synchronized to determine actual patient Hb levels. The indication for BGA followed clinical judgment and was not standardized across cases.

**Figure 3:**
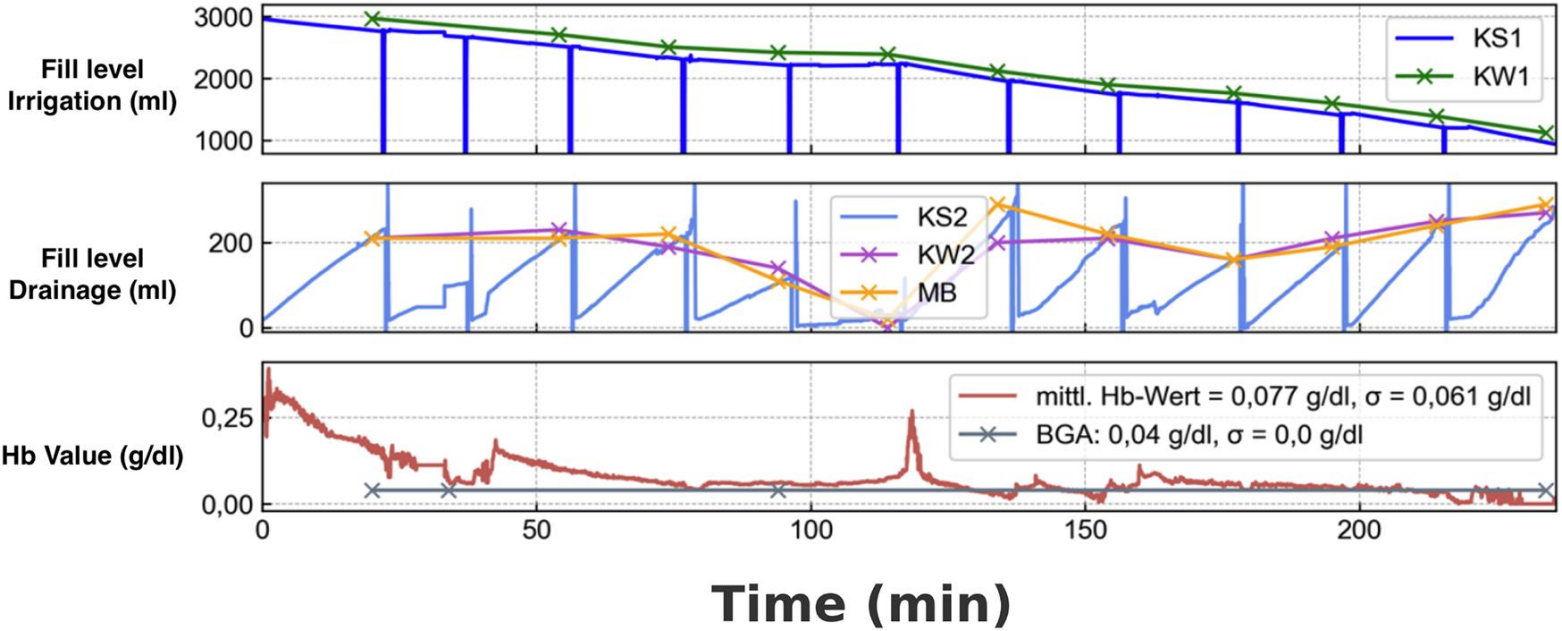
Comparison of sensor-based and manual measurements during continuous bladder irrigation monitoring. **(A)** Filling level of the irrigation bag (in mL): the blue curve (KS1) represents the sensor-based measurement of the irrigation bag, while the green curve shows the manual reference measurement using a scale. **(B)** Filling level of the drainage bag (in mL): the light blue curve displays the sensor-based measurement, the pink curve shows the manual measurement using a luggage scale, and the orange curve represents the cumulative volume measured manually with a graduated beaker. **(C)** Hemoglobin concentration over time: the red curve shows the sensor-measured hemoglobin concentration (average Hb value), while the gray curve indicates the reference measurements obtained by blood gas analysis (BGA) in g/dL.

All data collected by the VisIMon system, as well as the manually recorded reference values, were time-synchronized and subjected to descriptive graphical analysis. This included visualizing the course of measured Hb levels, fluid balances, and flow patterns over time (Figure 3). In addition to the technical validation, patient acceptance of the digital system was evaluated using a questionnaire. Patients were asked about their comfort with the system, whether they found it intrusive, and whether they preferred technical monitoring or conventional visual inspection by the clinical staff.

## 3 Results

**Figure 4:**
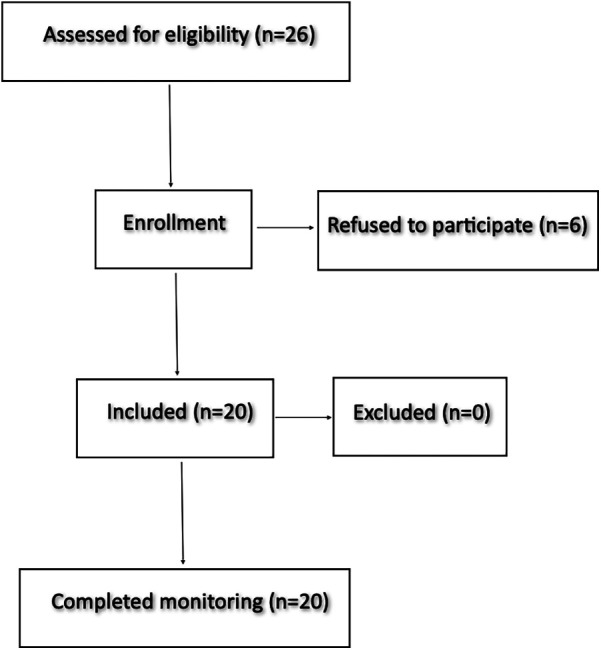
A total of 26 patients were screened for eligibility. Six patients declined participation, resulting in 20 enrolled subjects. All enrolled participants completed monitoring as planned. No participants were excluded.

All 20 patients enrolled in the study completed the monitoring period without adverse events related to the VisIMon system (Figure 4). The mean duration of continuous monitoring was 4.1 ± 0.3 h (Table 1). Throughout this period, the system continuously recorded the filling levels of the irrigation and drainage bags as well as the Hb concentration in the outflow. Data acquisition was stable and uninterrupted in all cases.

**Table 1:** Baseline demographic and clinical characteristics of the study cohort (*n* = 20). Data are presented as median (interquartile range) for continuous variables an *n* (%) for categorical variables.

Parameters	TURBT (*n* = 11)	TUR-P (*n* = 9)
Age (y)	65 (59–73)	69 (49–78)
Sex	Male 63%, Female 37%	Male 100%
Duration of CBI (h)	4 (3.9–4.05)	4.1 (4–4.2)
ASA	2.34 (2–3)	2.4 (2–3)

TURBT, transurethral resection of bladder tumor; TUR-P, transurethral resection of the prostate; CBI, continuous bladder irrigation; ASA, American society of anesthesiologists physical status classification

### 3.1 Accuracy of volume measurements

To evaluate the accuracy of the system's fluid volume detection, sensor-based measurements were compared to two independent reference methods: (1) volumetric determination using a measuring beaker and (2) weighing of the drainage bag at 20 min intervals. For the comparison with the measuring beaker, the VisIMon system showed a mean measured volume of 166.43 ± 80.00 mL (range: 7.58–300.00 mL), compared to 150.43 ± 73.99 mL (range: 4.00–300.00 mL) in the reference. The mean difference (bias) was +16.00 mL with a standard deviation of 31.30 ml and a 95% confidence interval for the bias of (9.87; 22.13) mL. The Pearson correlation coefficient between both measurements was *r* = 0.9203 [95% CI (0.8836; 0.9458)], indicating strong agreement. For the irrigation bag comparison, VisIMon measured a mean volume of 710.92 ± 822.25 mL (range: 0.00–3,000.00 mL), compared to 705.02 ± 823.01 mL (range: 0.00–3,000.00 mL) in the reference. The mean difference was +5.00 mL with a standard deviation of 25.77 mL and a 95% confidence interval for the bias of (0.84; 10.96) mL. The Pearson correlation coefficient was *r* = 0.9996 [95% CI (0.9993; 0.9998)], indicating near-perfect agreement. Sensor-derived values closely mirrored manually obtained data, demonstrating the system's ability to reliably detect changes in bladder irrigation volume. Short-term deviations in flow rate were observed in association with known disturbances such as drainage bag replacement or patient movement; these changes were transient and self-correcting.

### 3.2 Hemoglobin trend detection and comparison with BGA

The optical sensor component of the system detected variations in Hb concentration in the irrigation outflow over time. In patients with clinically suspected changes in hematuria severity, BGA was performed at the discretion of the attending physician. While the number and timing of BGA measurements were not standardized, available data showed a qualitative correlation between the Hb trends captured by the VisIMon system and the corresponding BGA values. The mean deviation between the system's optical Hb measurement and BGA reference values was −0.003 g/dL, indicating strong concordance. Descriptive analysis further confirmed close agreement between both methods. For the sensor-based Hb values, the minimum was 0.004 g/dL, the maximum 3.197 g/dL, the mean 0.204 g/dL with a standard deviation of ±0.600 g/dL, and the 95% confidence interval ranged from 0.033–0.374 g/dL. For the BGA reference values, the minimum was 0.007 g/dL, the maximum 3.200 g/dL, the mean 0.207 g/dL, with the same standard deviation of ±0.600 g/dL and a 95% confidence interval of 0.036–0.377 g/dL. The Pearson correlation coefficient between paired measurements was approximately *r* = 1.00, indicating nearly perfect linear agreement. For example, in patients with an increasing Hb trend detected by the system, BGA frequently confirmed a rising systemic Hb level in the drainage, suggesting active bleeding.

### 3.3 Detection of catheter-associated events

In 10 cases, the system identified abrupt changes in flow patterns, such as sudden stagnation of outflow or irregularities consistent with the presence of air bubbles. These events were logged and visually displayed on the user interface. Clinical confirmation during ward rounds verified the presence of transient flow obstructions, for example due to kinking of the catheter or drainage tubing. One relevant complication occurred due to incorrect placement of the optical sensor: at the beginning of the monitoring period, the irrigation tubing was twisted, which temporarily affected the measurement accuracy until the setup was corrected. Overall, the system proved capable of recognizing mechanical disturbances in real time, offering potential for earlier clinical intervention.

### 3.4 Patient safety and usability

**Figure 5:**
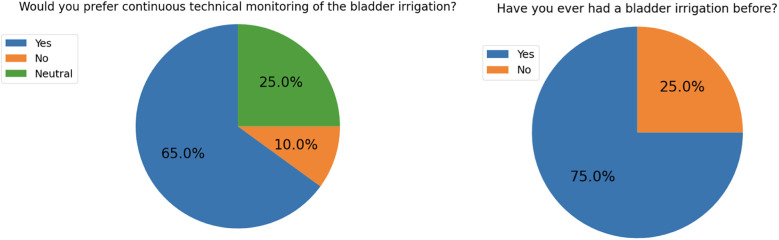
Results of the patient survey conducted after the application of the VisIMon irrigation monitoring system. Seventy-five percent of patients reported no prior experience with bladder irrigation. The majority of patients preferred continuous technical monitoring of the irrigation process, whereas only 10% favored traditional visual inspection. Twenty-five percent of respondents indicated no clear preference.

All patients tolerated the system well, and no study terminations were necessary due to discomfort or technical malfunction. The wearable components, including the sensor mounts, remained in place throughout the monitoring period. The system functioned reliably in routine clinical settings, and nursing staff were able to interact with the interface without additional training beyond the initial introduction. Patient feedback was largely positive: the majority reported that the system increased their perceived sense of safety without causing discomfort. Specifically, 13 of 20 patients preferred the digital monitoring system over manual observation, 5 expressed no preference, and only 2 favored traditional visual inspection (Figure 5).

### 3.5 Graphical visualization of clinical parameters

The integration of system data into graphical time-series visualizations enabled comprehensive analysis of irrigation behavior. For each patient, the time-aligned plots of irrigation inflow, outflow, and Hb concentration allowed intuitive interpretation of clinical dynamics and correlation with reference data. These visualizations revealed the interplay between irrigation volume and bleeding trends and illustrated the effect of clinical interventions such as catheter flushing or bag replacement.

### 3.6 Feasibility outcomes

The feasibility of the VisIMon system was evaluated based on accuracy during dynamic conditions, system stability, and patient acceptance. First, the system demonstrated high fidelity in reflecting true physiological changes. Across all patients, sensor-derived measurements for irrigation volume and hemoglobin concentration showed strong concordance with reference standards, with Pearson correlation coefficients consistently exceeding 0.9. This agreement was preserved even during dynamic phases of the postoperative course, such as active bleeding or temporary outflow obstruction, demonstrating the system's robustness under clinically relevant conditions. Second, system uptime was high. Across all 20 patients, a total of 50 short-lived measurement interruptions occurred, corresponding to an average of 2.5 interruptions per patient. Each interruption lasted less than one minute, and no case required switching back to manual monitoring. Over the standard 4-hour observation period (240 min), this corresponded to an effective data acquisition time of approximately 99%. Third, patient acceptance was high. Based on structured postoperative interviews, only 2 out of 20 patients (10%) expressed a preference for traditional visual control and opted against the digital system in the future. No patient reported a subjective reduction in safety or comfort due to the use of the sensor (Table 2).

**Table 2:** Feasibility metrics of the VisIMon system during continuous bladder irrigation monitoring across 20 postoperative patients. The table summarizes key indicators of technical accuracy, system stability, and patient acceptance. Sensor data showed high correlation with reference measurements, minimal short-lived interruptions, and high overall user acceptability.

Dimension	Indicator	Result	Comment
Technical accuracy	Pearson correlation with reference methods	*r* > 0.90	High accuracy even during bleeding episodes or outflow issues
	Qualitative agreement in dynamic conditions	Consistent trend tracking	Hb and flow changes mirrored clinical course
Data stability	Total number of sensor interruptions	50 events across all patients	∅ 2.5 per patient; no critical failures
	Duration of interruptions	<1 min each	Self-resolving, transient
	Overall data acquisition uptime (4 h monitoring period)	∼99%	Continuous monitoring maintained
Patient acceptance	Patients preferring visual/manual over sensor-based monitoring	2/20 patients (10%)	Majority favored digital system
	Subjective safety concerns reported	None	No adverse patient feedback

## 4 Discussion

In this prospective feasibility study, we demonstrated that the VisIMon digital monitoring system enabled continuous, reliable, and accurate surveillance of CBI following transurethral surgery. The system showed high concordance with manual reference measurements for irrigation volumes and Hb concentrations, while successfully detecting clinically relevant catheter-associated events such as drainage obstructions and air bubbles. Importantly, the VisIMon system was well tolerated by patients and received positive usability feedback from clinical staff, suggesting high acceptability in a real-world postoperative setting.

The findings of this study suggest that sensor-based real-time monitoring of CBI can significantly enhance postoperative urological care. Continuous and objective recording of bladder filling levels, irrigation volumes, and Hb concentrations may also increase patient safety and improve clinical workflow efficiency. Compared to conventional intermittent manual inspections, digital monitoring allows earlier detection of complications such as catheter obstructions, bladder overdistension, or active bleeding. The availability of real-time, quantifiable data facilitates faster clinical decision-making, potentially reducing adverse events and optimizing postoperative outcomes.

Previous technical approaches have pursued similar goals of automating bladder irrigation monitoring. Ding et al. (2016) described the development of a wireless sensor-based system for automated regulation of CBI, demonstrating feasibility in a clinical setting (14). Similarly, Chan et al. (2020) presented a laboratory-based proof-of-concept system capable of detecting gross hematuria and providing automated feedback (15). More recently, Tao et al. (2024) reported on a self-improving sensor device for CBI, showing that its use could enhance patients' disease awareness, reduce anxiety, improve compliance, and lower complication rates following TURP (16). Compared to these earlier efforts, VisIMon offers a more comprehensive solution by combining volumetric monitoring, optical hemoglobin detection, automated event recognition, and real-time visualization, thereby actively supporting clinical workflows.

Other developments in bladder sensor technology have predominantly focused on non-invasive or implantable systems designed to monitor bladder volume. Various approaches such as bed-based weight sensor systems (17), wearable ultrasound devices (18, 19), near-infrared spectroscopy sensors (20), and bioimpedance-based monitoring platforms (21) have demonstrated promising results for estimating urinary bladder filling levels. These technologies aim to improve continence care, reduce catheterization rates, and enhance the management of neurogenic bladder dysfunctions. However, the primary focus of these innovations has been volumetric assessment, often without real-time analysis of fluid characteristics. In contrast, the VisIMon system extends beyond pure volumetric monitoring by incorporating continuous optical Hb concentration measurement in the drainage fluid. This unique combination enables not only precise fluid balance surveillance but also dynamic assessment of bleeding severity, offering a more comprehensive and clinically actionable real-time overview during continuous bladder irrigation.

From a clinical usability standpoint, the system was well tolerated by patients and easily adopted by clinical staff. No adverse events were reported, and there were no dropouts due to technical or user-related issues. The graphical visualization of synchronized parameters (inflow, outflow, Hb) was especially appreciated by the care team, offering an at-a-glance overview of the patient's status and reducing the need for repeated bedside visits.

The implementation of the VisIMon system introduces several important advantages into daily clinical practice. Precise volume monitoring facilitates more accurate planning of irrigation bag changes, minimizing the risk of unintentional overflow. Objective, real-time assessment of drainage fluid coloration enhances the detection of changes in hematuria severity compared to subjective visual inspections. Automatic alerts for irrigation interruptions or mechanical obstructions allow prompt clinical interventions. Moreover, the system's ability to display data both centrally at nurse stations and peripherally on mobile devices improves information accessibility. Continuous digital recording of irrigation dynamics also supports fluid balance documentation and treatment quality control, offering new opportunities for structured postoperative care.

By automating routine monitoring tasks, the VisIMon system significantly reduces the workload on nursing staff, particularly during night shifts or in high-patient-load scenarios. This efficiency gain can lead to optimized resource allocation, enabling staff to focus on more complex patient care needs. Furthermore, early detection of complications may prevent costly secondary interventions or prolonged hospital stays. The system's full digital documentation facilitates patient-specific billing, provides transparent treatment records for internal quality assurance, and supports compliance with external healthcare reporting standards. Overall, these features can contribute to both improved clinical outcomes and cost savings for healthcare institutions.

The next generation of the VisIMon system, now commercialized as filaxONE, further expands clinical integration opportunities (22)^1^. FilaxONE enables automated recording of CBI events, real-time detection of fluid coloration, and network-independent Wi-Fi operation with end-to-end encrypted communication. Integration into hospital information systems (HIS) is possible through standardized interfaces, with options for scalable, ISO/IEC 27001-certified cloud infrastructure or on-premise solutions. These features ensure that all data, including fluid balances and catheter performance metrics, are automatically stored within electronic health records (EHRs), enhancing documentation quality and traceability. Centralized and decentralized visualization supports flexible clinical workflows. In the future, this integration could enable fully automated, closed-loop irrigation systems based on continuous sensor feedback.

Beyond technical integration, the broader implementation also depends on economic and regulatory considerations. Although a formal cost-effectiveness analysis was not performed in this feasibility phase, the system's potential to reduce nursing workload, prevent avoidable complications, and streamline documentation processes may yield significant economic benefits. These include lower staff time per patient, fewer catheter-related re-interventions, and improved case-based billing accuracy due to real-time fluid tracking. From a regulatory perspective, filaxONE is currently being developed in accordance with the European Medical Device Regulation. The system includes CE-marked components, end-to-end encrypted communication, and optional ISO/ IEC 27001-certified cloud infrastructure. Integration into hospital environments is supported via standardized data interfaces and ongoing conformity assessments. These features ensure that clinical deployment can proceed in line with current safety, data, and documentation regulations.

However, despite the system's technical readiness for clinical integration—including support for Wi-Fi operation, EHR connectivity, and certified data infrastructure—the practical implementation within hospital environments still poses notable challenges. These include interoperability with heterogeneous HIS environments, alignment with hospital-specific IT policies, and compliance with data governance frameworks. Even if ISO/IEC 27001-compliant infrastructure is technically available, actual deployment often requires detailed review by institutional data protection officers and local IT governance boards. Beyond technical integration, organizational aspects such as assignment of monitoring responsibilities, staff training, and alert management must be addressed. Clinical feedback from our pilot application emphasized the importance of an intuitive user interface, low-threshold learning curves, and seamless workflow integration—particularly during high workload periods or night shifts. To enable sustainable deployment, future implementation studies will need to evaluate not only system performance, but also process-level adjustments and institutional prerequisites for routine use. This includes staff readiness, documentation policies, and decision-making protocols linked to sensor-based alerts.

This study has several limitations. First, the sample size was relatively small (*n* = 20), and the observation period was limited to approximately four hours postoperatively. As a result, the findings primarily reflect short-term feasibility rather than long-term clinical performance. Second, BGA measurements were performed at the discretion of the clinical team rather than according to a standardized protocol, limiting quantitative correlation analyses. This decision reflects both resource considerations and the need for time-sensitive detection of hematuria dynamics. Future studies will implement standardized BGA reference measurements to enable quantitative validation while preserving the system's strength in continuous monitoring. Third, the system used was an early prototype with technical limitations, including occasional challenges with sensor fixation and restricted integration with hospital information systems. Fourth, as the system was not yet certified as a medical device at the time of the study, no predefined hemoglobin thresholds for clinical alarm triggering were implemented. The monitoring interface remained observational only, without influencing clinical decision-making, in accordance with the restrictions imposed by the ethics committee. Finally, the study design lacked a parallel control group receiving conventional manual CBI monitoring. Without a comparative cohort, it is not possible to formally assess the relative advantages of VisIMon over standard care. To establish generalizability and assess the comparative clinical benefits of VisIMon, larger randomized controlled trials with longer observation periods and standard-of-care control arms will be essential.

## 5 Conclusion

In summary, the VisIMon system demonstrated technical feasibility, clinical reliability, and high user acceptance in the continuous monitoring of CBI following transurethral urological procedures. By improving patient safety, optimizing clinical workflows, and enabling seamless digital documentation, the system represents a promising innovation in postoperative urological care. Future studies should focus on long-term outcomes, cost-effectiveness analyses, and the development of fully automated, closed-loop irrigation solutions.

## Data Availability

The datasets presented in this article are not readily available due to data protection policies. Requests to access the datasets should be directed to the corresponding author.

## Footnotes

1
https://filax-medical.com
